# Closing Editorial: Composition and Biophysical Properties of Lipid Membranes

**DOI:** 10.3390/membranes16040143

**Published:** 2026-04-07

**Authors:** Mariana Ferreira

**Affiliations:** 1 LAQV/REQUIMTE (Laboratório Associado Para a Química Verde—Rede de Química e Tecnologia), Departamento de Química e Bioquímica, Faculdade de Ciências, Universidade do Porto, Rua do Campo Alegre, s/n, 4169-007 Porto, Portugal; mariana.ferreira@fc.up.pt or sre@ess.ipp.pt; 2 Chemical and Biomolecular Sciences, E2S, Polytechnic of Porto, R. Dr. António Bernardino de Almeida, 400, 4200-072 Porto, Portugal

## 1. Introduction

Biological membranes are essential structural and functional components of living systems, forming the boundaries that define cells and organelles while enabling selective transport, signalling, and energy transduction [1]. Their fundamental architecture—a lipid bilayer embedded with proteins—creates a dynamic interface whose physical properties are strongly influenced by lipid composition. Variations in lipid headgroups, acyl chain length, and degree of saturation determine important membrane characteristics such as fluidity, curvature, permeability, and mechanical stability. Understanding how these compositional factors govern membrane behaviour remains a central objective in membrane biophysics and continues to drive interdisciplinary research [2,3].

Over the past few decades, major methodological advances have significantly improved our ability to study lipid membranes. Experimental techniques such as fluorescence spectroscopy, calorimetry, scattering methods, and high-resolution microscopy have enabled detailed characterization of membrane organization and phase behaviour [4,5,6,7,8,9,10]. At the same time, computational approaches—including molecular dynamics simulations—have become powerful tools for exploring the molecular mechanisms underlying membrane structure, lipid–protein interactions, and transport processes [2,11,12,13,14]. Together, these approaches have contributed to important insights into membrane elasticity, lipid phase transitions, and nanoscale domain formation [15,16,17,18].

## 2. Challenges

Despite this progress, several fundamental challenges remain. Biological membranes display remarkable compositional diversity, often containing hundreds of lipid species that are asymmetrically distributed between bilayer leaflets. This heterogeneity generates emergent physical properties that are difficult to reproduce in simplified model systems. In addition, the interplay between lipid composition, membrane curvature, and protein activity introduces further complexity in processes such as membrane remodelling, fusion, and cellular signalling. Bridging the gap between well-controlled model membranes and the complex environment of living cells remains an important goal in membrane research.

Another challenge lies in integrating knowledge across multiple spatial and temporal scales. Experimental techniques can provide detailed structural and thermodynamic information about membranes, but they often capture only specific aspects of membrane dynamics. Conversely, computational simulations offer molecular-level mechanistic insights but remain limited by system size and accessible timescales. Therefore, a comprehensive understanding of membrane behaviour requires integrative approaches that combine experimental measurements, theoretical frameworks, and computational modelling.

## 3. Overview of Special Issue Contributions

The Special Issue “Composition and Biophysical Properties of Lipid Membranes” was established to provide a platform for diverse studies examining how lipid composition influences membrane structure, dynamics, and interactions. The ten collected papers reflect the breadth of contemporary membrane research and encompass experimental investigations, theoretical analyses, and computational approaches.

Several contributions focus on the physicochemical properties of lipid bilayers and their dependence on molecular composition, providing insights into how specific lipid components influence membrane stability, phase behaviour, and mechanical properties, and thereby helping to clarify the fundamental relationships between lipid structure and bilayer organization. Investigations employing model membrane systems highlight how controlled lipid compositions can be used to dissect the physical principles governing membrane organization and dynamics.

Other articles included in this Special Issue explore interactions between lipid membranes and biologically active molecules. For instance, recent work has examined how mitochondrial membrane composition affects the aggregation and membrane interactions of amyloidogenic peptides, illustrating the critical role lipid environments play in modulating peptide-induced membrane damage [19]. This underscores the importance of membrane composition in processes relevant to disease and cellular stress responses.

In addition, several contributions investigate transport processes and membrane permeability. Theoretical analyses of osmotic permeabilities in aquaglyceroporins—including AQP7, AQP10, and the bacterial channel GlpF—have provided insights into the molecular mechanisms governing water transport through biological membranes [20]. Such work advances our understanding of selective transport across lipid bilayers and membrane channels.

This Special Issue also highlights how membrane properties respond to external physical or chemical perturbations. Investigations addressing membranes’ responses to oxidative environments or other stress conditions illustrate how environmental factors can influence bilayer structure and stability, providing insights into processes such as membrane disruption and stress-induced remodelling [21].

Taken together, the selected contributions demonstrate the value of combining complementary methodologies to investigate membrane phenomena from multiple perspectives. By integrating experimental observations with theoretical and computational analyses, these studies provide new insights into how lipid composition governs membrane biophysics while emphasizing the continued relevance of model membrane systems for studying complex biological processes.

Looking forward, several research directions appear particularly promising. The development of multi-scale approaches linking molecular simulations with experimentally measurable membrane properties will be essential for bridging the gap between molecular mechanisms and cellular behaviour. Advances in lipidomics and super-resolution imaging are expected to reveal new details about lipid organization and dynamics within living membranes. Furthermore, experimental models that better reproduce membrane asymmetry and compositional complexity will enhance the biological relevance of membrane studies.

Beyond fundamental research, the intersection of membrane biophysics with emerging fields such as synthetic biology, nanomedicine, and biomaterials engineering offers exciting opportunities. The design of artificial membranes with tailored composition and mechanical properties may enable the development of advanced biomimetic materials, biosensors, and lipid-based drug delivery systems.

## 4. Conclusions

In conclusion, the studies presented in this Special Issue highlight the progress achieved in understanding the relationships between lipid composition and membrane biophysics while also highlighting important questions that have yet to be addressed. By bringing together diverse approaches and perspectives, this collection helps to deepen our understanding of the physical principles governing biological membranes and will hopefully stimulate further research in this dynamic field.

## References

[1] Watson H. (2015). Biological membranes. Essays Biochem..

[2] Muller M.P., Jiang T., Sun C., Lihan M., Pant S., Mahinthichaichan P., Trifan A., Tajkhorshid E. (2019). Characterization of Lipid-Protein Interactions and Lipid-Mediated Modulation of Membrane Protein Function through Molecular Simulation. Chem. Rev..

[3] Friedman R., Khalid S., Aponte-Santamaría C., Arutyunova E., Becker M., Boyd K.J., Christensen M., Coimbra J.T.S., Concilio S., Daday C. (2018). Understanding Conformational Dynamics of Complex Lipid Mixtures Relevant to Biology. J. Membr. Biol..

[4] Ferreira A.R., Ferreira M., Nunes C., Reis S., Teixeira C., Gomes P., Gameiro P. (2023). The Unusual Aggregation and Fusion Activity of the Antimicrobial Peptide W-BP100 in Anionic Vesicles. Membranes.

[5] Loura L.M.S., Prieto M., Roberts G.C.K. (2013). Fluorescence and FRET in Membranes. Encyclopedia of Biophysics.

[6] de Almeida R.F., Loura L.M., Prieto M. (2009). Membrane lipid domains and rafts: Current applications of fluorescence lifetime spectroscopy and imaging. Chem. Phys. Lipids.

[7] Okamoto Y., Hamaguchi K., Watanabe M., Watanabe N., Umakoshi H. (2022). Characterization of Phase Separated Planar Lipid Bilayer Membrane by Fluorescence Ratio Imaging and Scanning Probe Microscope. Membranes.

[8] Michel N., Fabiano A.-S., Polidori A., Jack R., Pucci B. (2006). Determination of phase transition temperatures of lipids by light scattering. Chem. Phys. Lipids.

[9] Montenegro L., Castelli F., Sarpietro M.G. (2018). Differential Scanning Calorimetry Analyses of Idebenone-Loaded Solid Lipid Nanoparticles Interactions with a Model of Bio-Membrane: A Comparison with In Vitro Skin Permeation Data. Pharmaceuticals.

[10] Lopes S., Neves C.S., Eaton P., Gameiro P. (2010). Cardiolipin, a key component to mimic the E. coli bacterial membrane in model systems revealed by dynamic light scattering and steady-state fluorescence anisotropy. Anal. Bioanal. Chem..

[11] Boulos I., Jabbour J., Khoury S., Mikhael N., Tishkova V., Candoni N., Ghadieh H.E., Veesler S., Bassim Y., Azar S. (2023). Exploring the World of Membrane Proteins: Techniques and Methods for Understanding Structure, Function, and Dynamics. Molecules.

[12] Majeed S., Ahmad A.B., Sehar U., Georgieva E.R. (2021). Lipid Membrane Mimetics in Functional and Structural Studies of Integral Membrane Proteins. Membranes.

[13] Venable R.M., Krämer A., Pastor R.W. (2019). Molecular Dynamics Simulations of Membrane Permeability. Chem. Rev..

[14] Magalhães B.T., Coimbra J.T.S., Silva R.M., Ferreira M., Santos R.S., Gameiro P., Azevedo N.F., Fernandes P.A. (2024). Crosstalk of Nucleic Acid Mimics with Lipid Membranes: A Multifaceted Computational and Experimental Study. Biochemistry.

[15] Stelter D., Keyes T. (2022). Membrane Phase Transitions in Lipid-Wrapped Nanoparticles. J. Phys. Chem. B.

[16] Woodward X., Javanainen M., Fábián B., Kelly C.V. (2023). Nanoscale membrane curvature sorts lipid phases and alters lipid diffusion. Biophys. J..

[17] Renard K., Byrne B. (2021). Insights into the Role of Membrane Lipids in the Structure, Function and Regulation of Integral Membrane Proteins. Int. J. Mol. Sci..

[18] Yamada T., Shinoda W. (2026). Asymmetry and heterogeneity in the plasma membrane. Biophys. J..

[19] Seychell R.M., El Saghir A., Farrugia G., Vassallo N. (2025). Coenzyme Q10 Enhances Resilience of Mitochondrial-like Membranes Against Amyloidogenic Peptides. Membranes.

[20] Chan R., Chen L.Y. (2025). Application of the Transition State Theory in the Study of the Osmotic Permeabilities of AQP7, AQP10 and GlpF. Membranes.

[21] de Carvalho J.C.S., Nobre-Azevedo P., da Silva-Neto P.V., Oliveira B.T.M., Tavares L.A., Toro D.M., Borges A.O., Nascimento M.A., Arruda E., Martins R.B. (2026). Fluoxetine Reshapes Macrophage Membrane Sphingolipids and Inflammatory Response Without Affecting Extracellular Vesicle Biogenesis upon Inactivated SARS-CoV-2 Stimulation. Membranes.

